# Risk Assessment of Deoxynivalenol by Revisiting Its Bioavailability in Pig and Rat Models to Establish Which Is More Suitable

**DOI:** 10.3390/toxins7124873

**Published:** 2015-12-01

**Authors:** Manuel Jimmy Saint-Cyr, Agnès Perrin-Guyomard, Jacqueline Manceau, Paméla Houée, Jean-Michel Delmas, Jean-Guy Rolland, Michel Laurentie

**Affiliations:** 1 ANSES, Fougères Laboratory, Antibiotics, Biocides, Residues and Resistance Unit, 10B rue Claude Bourgelat, Javené, CS 40608, Fougères 35306, France; agnes.perrin-guyomard@anses.fr (A.P.-G.); pamela.houee@anses.fr (P.H.); 2 ANSES, Fougères Laboratory, Scientific Support Unit, 10B rue Claude Bourgelat, Javené, CS 40608, Fougères 35306, France; jacqueline.manceau@anses.fr (J.M.); jean-guy.rolland@anses.fr (J.-G.R.); michel.laurentie@anses.fr (M.L.); 3 ANSES, Fougères Laboratory, Analysis of Residues and Contaminants Unit, 10B rue Claude Bourgelat, Javené, CS 40608, Fougères 35306, France; jean-michel.delmas@anses.fr

**Keywords:** deoxynivalenol, bioavailability, toxicokinetic, risk assessment

## Abstract

Due to its toxic properties, high stability, and prevalence, the presence of deoxynivalenol (DON) in the food chain is a major threat to food safety and therefore a health risk for both humans and animals. In this study, experiments were carried out with sows and female rats to examine the kinetics of DON after intravenous and oral administration at 100 µg/kg of body weight. After intravenous administration of DON in pigs, a two-compartment model with rapid initial distribution (0.030 ± 0.019 h) followed by a slower terminal elimination phase (1.53 ± 0.54 h) was fitted to the concentration profile of DON in pig plasma. In rats, a short elimination half-life (0.46 h) and a clearance of 2.59 L/h/kg were estimated by sparse sampling non-compartmental analysis. Following oral exposure, DON was rapidly absorbed and reached maximal plasma concentrations (*C*_max_) of 42.07 ± 8.48 and 10.44 ± 5.87 µg/L plasma after (*t*_max_) 1.44 ± 0.52 and 0.17 h in pigs and rats, respectively. The mean bioavailability of DON was 70.5% ± 25.6% for pigs and 47.3% for rats. In the framework of DON risk assessment, these two animal models could be useful in an exposure scenario in two different ways because of their different bioavailability.

## 1. Introduction

Deoxynivalenol (DON) is a secondary fungal metabolite of the trichothecene family. Produced by Fusarium species, it is one of the most prevalent mycotoxins in cereal crops worldwide, and the most frequently occurring type B trichothecene in Europe. A large-scale data survey indicated that DON is present in 43.5% of food and 75.2% of feed samples collected in the European Union [1]. Epidemiological studies linking DON exposure to adverse health effects in humans have been reported in China, India, Japan, and Korea [2,3,4]. DON-contaminated foods cause human gastroenteritis with nausea, diarrhea, and vomiting. In addition to the symptoms described in humans, DON toxicity in animals leads them to refuse feed, with consequent growth retardation [5]. At the cellular level, even though DON poses no genotoxic or mutagenic risk [6,7], it has been shown to inhibit protein synthesis and modulate immune responses [8,9]. Due to its toxic properties, high stability, and prevalence, the presence of DON in the food chain is a major threat to food safety, representing a health risk for both humans and animals [10,11,12]. This risk was characterized in animals by toxicological, epidemiological and kinetic studies reviewed by Pestka (2007) [5]. A No Observable Adverse Effect Level (NOAEL) has been established at 100 μg/kg of body weight (bw) based on a decreased body weight gain reported in a two-year feeding study in mice [13].

Despite its well-known toxicological effects and established epidemiological data, information on DON kinetics is more limited, although knowledge of the kinetic parameters of a toxic agent like DON is essential for evaluating animal and human health risks. In addition, oral bioavailability—a major parameter—has rarely been defined, despite DON being an orally-ingested food contaminant. Furthermore, the few kinetic studies that are available have focused on farm animals such as chickens, pigs, or sheep [14,15,16,17,18], whereas toxicological studies have focused on rodents [19] in order to link dose and effects. Moreover, the parameters defined could be misused and/or unsuitable for two reasons: first, the use of data below the limit of quantification for kinetic analysis, and second, the use of an unsuitable approach to estimating kinetic parameters by misuse of the mean and conventional kinetic approach through sparse sampling. In addition, these studies are not recent, and new insights need to be sought.

The aim of this study was to assess DON toxicokinetics in two animal models after intravenous (IV) and oral gavage at the NOAEL dose. We used pigs because their vascular anatomy and physiology are similar to humans, and rats because they are conventionally used for toxicological studies. The parameters thus obtained may help to improve knowledge on DON kinetics at NOAEL.

## 2. Results

### 2.1. Clinical Signs

Deoxynivalenol was tolerated by all animals, but there was an obvious difference in the manifestation of side effects when comparing species or ways of administration. All pigs administered intravenously with 100 µg DON/kg bw showed signs of acute DON toxicosis, salivation, retching, and emesis within a few minutes (8–15 min). In contrast, at the same dose, no signs of toxicity were observed in pigs following oral administration, or in rats irrespective of the route of administration.

### 2.2. Concentration Profile after IV Administration of DON

**Figure 1:**
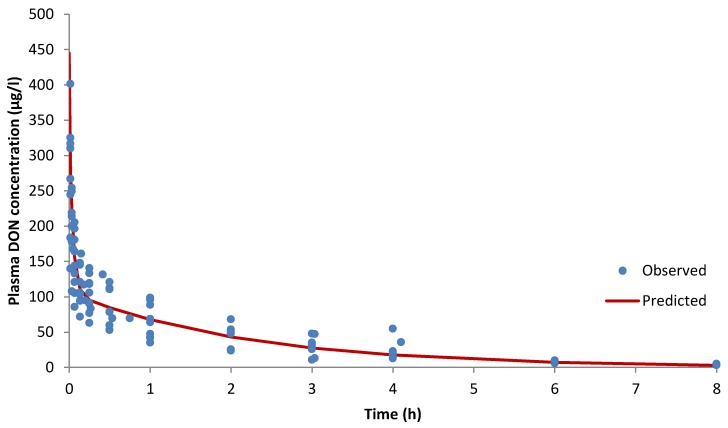
Two-compartment model fitted to DON concentrations (µg/L) *vs.* time (h) in plasma in pigs after IV administration of 100 µg/kg of DON. (Full circle is observed data; solid line is predicted data).

Following intravenous dosing, a two-compartment model was fitted to the concentration profile of DON in pig plasma based on Akaike’s information criterion (Figure 1) [20].

This two-compartment model is described by the bi-exponential equation below:

(1) $$C\left(t\right)=Ae^{-\alpha t}+Be^{-\beta t}$$

where *C*(*t*) is the DON concentration at time t; A and B the extrapolated values to time 0 of the first and second phases of DON concentration disposition and α, β the rate constant of distribution and elimination phases, respectively. From Equation (4), kinetic parameters were calculated and reported in Table 1. Initial distribution was rapid, with a mean half-life (*t*_1/2_α) of 0.030 ± 0.019 h, followed by a slower terminal elimination phase (*t*_1/2_β) of 1.53 ± 0.54 h (Table 1). The total plasma clearance (Cl) of DON calculated from the equation described by Toutain and Bousquet-Mélou (2004) [21] was 0.42 ± 0.17 L/h/kg (Table 1). The volume of distribution at steady state was equal to 0.88 ± 0.17 L/kg.

The non-compartmental analysis (NCA) (Table 2) confirmed the results obtained with the compartmental analysis for the different estimated parameters. No statistical differences were observed between parameters. MRT was estimated at 2.24 ± 1.15 h.

**Table 1:** Individual and mean toxicokinetic parameters of DON estimated from a two-compartment model in the plasma of seven pigs following intravenous administration of a single dose of 100 µg/kg.

Parameters (Units)	1	2	3	4	5	6	7	Mean	SD
**A (µg/L)**	2.41	2.57	1.53	2.14	1.48	4.76	2.68	2.51	1.10
**B (µg/L)**	1.19	1.46	0.99	0.62	1.03	1.00	1.15	1.06	0.26
**α (1/h)**	13.86	30.87	21.86	8.16	25.47	48.85	14.24	23.33	13.65
**β (1/h)**	0.35	0.52	0.38	0.43	0.47	0.74	0.27	0.45	0.15
**AUC_INF_ (h·µg/L)**	354.64	290.11	264.71	168.71	224.92	144.07	480.64	275.40	115.41
***t*_1/2_α (h)**	0.050	0.022	0.032	0.085	0.027	0.014	0.049	0.030 ^a^	0.019
***t*_1/2_β (h)**	1.97	1.33	1.81	1.61	1.48	0.93	2.56	1.53 ^a^	0.54
**Cl (L/h/kg)**	0.28	0.34	0.38	0.59	0.44	0.69	0.22	0.42	0.17
**MRT_INF_ (h)**	2.70	1.87	2.55	1.98	2.08	1.26	3.54	2.28	0.73
**Vss (L/kg)**	0.76	0.64	0.96	1.17	0.92	0.87	0.80	0.88	0.17

A: extrapolated zero-time plasma DON concentration in the α phase; B: extrapolated zero-time plasma DON concentration in the β phase; α: distribution rate constant; β: elimination rate constant; *t*_1/2_: biological half-life of α (distribution) or β (elimination); AUC_INF_: area under the curve; Cl: clearance; MRT_INF_: mean residence time; Vss: volume of distribution at steady state. ^a^: mean is a harmonic mean with its SD.

**Table 2:** Comparison of toxicokinetic parameters of DON determined by non-compartmental analysis after intravenous or oral administration in rats and pigs.

Parameters (Units)	Pigs		Rats	
	IV	Gavage	IV	Gavage
**Lambda_z (1/h)**	0.43 ± 0.21	0.29 ± 0.16	1.51	0.18
**HL_Lambda_z (h)**	1.49± 0.64 ^a^	2.38 ± 1.45 ^a^	0.46	3.95
**AUC_last_ (h·µg/L)**	222.3 ± 106.72	120.5 ± 29.88	30.91 ± 7.87	14.63 ± 4.45
**AUC_INF_ (h·µg/L)**	253.8 ± 123.33	197.2 ± 88.50	38.65	48.81
**AUC_Extrap_ (%)**	9.76 ± 2.64	33.79 ± 17.60	20.03	70.03
**Cl (L/h/kg)**	0.41 ± 0.17	-	2.590	-
**E_body_**	0.07	-	0.17	-
**MRT_last_ (h)**	1.41 ± 0.61	2.43 ± 0.59	0.32	0.95
**MRT_INF_ (h)**	2.24 ± 1.15	5.59 ± 4.01	0.58	5.68
**Vss (L/kg)**	0.78 ± 0.17	-	1.51	-
***t*_max_ (h)**	-	1.44 ± 0.52	-	0.17
***C*_max_ (µg/L)**	-	42.07 ± 8.48	-	10.44 ± 5.87

Results were expressed as the mean of the parameter (*n* = 7 pigs) ± standard deviation. Lambda_z: first order rate constant associated with the terminal (log-linear) portion of the curve; HL_Lambda_z: terminal half-life (ln(2)/terminal slope); AUC_last_: Area Under the Curve (AUC) from time of dosing (0) to the time of the last quantifiable concentration (*i.e.*, above LOQ); AUC_INF_: AUC extrapolated from time of dosing (0) to infinity; AUC_Extrap_: Percentage of AUC_INF_ that is due to extrapolation from t_last_ to infinity; Cl: clearance; E_body_: body extraction ratio; MRT_last_: Mean Residence Time (MRT) from time of dosing to the last quantifiable concentration; MRT_INF_: MRT extrapolated to infinity using the last quantifiable concentration for extrapolation; Vss: volume of distribution at steady state; *t*_max_: time of maximum plasma DON concentration; *C*_max_: maximum plasma DON level. ^a^: harmonic mean with its SD.

**Figure 2:**
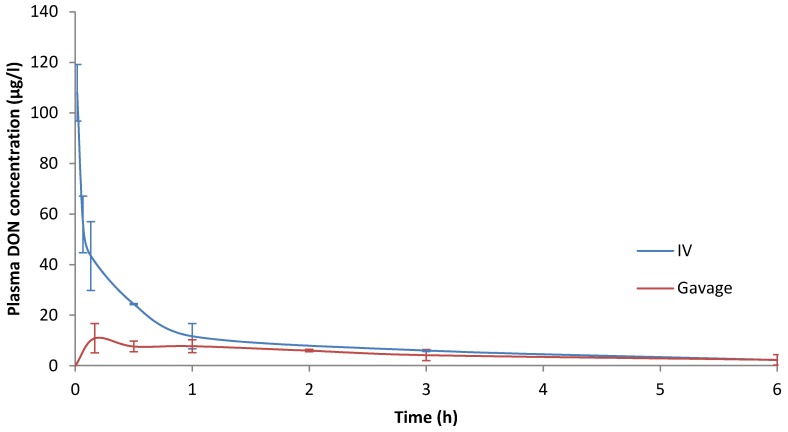
Time course evolution of mean DON concentrations (µg/L) *vs.* time (h) in rat plasma after IV or oral administration of 100 µg/kg of DON (*n* = 3 per sampling time).

In rats, we assessed the kinetic parameters of DON after IV administration by sparse sampling NCA. The results obtained are presented in Table 2 and the time course evolution of mean DON concentrations in plasma is illustrated in Figure 2.

A short elimination half-life (0.46 h), clearance of 2.59 L/h/kg and Vss of 1.5 L/kg were estimated. The MRT was also short, with values of 0.32 h to 0.58 h for MRT_last_ and MRT_INF_, respectively. It was also observed that the extrapolated area makes a major contribution (20%) to the total area (AUC_INF_).

To compare clearance of rat and pigs, the body extraction ratio (E_body_) was determined from cardiac output. E_body_ and cardiac output were calculated with equations classically described [21] with a hepatic and renal extraction ratio equal to 1. Under these conditions, we estimated that E_body_ was 0.07 for pigs and 0.17 for rats. From the reference values provided for E_body_, a value close to 0.05 indicates poor clearance and a value close to 0.15 moderate clearance. Consequently, the clearance of DON in pigs is poor, whereas in rats it is moderate. Furthermore, clearance is three times higher in rats than in pigs.

### 2.3. Concentration Profile after Oral Administration of DON

**Figure 3:**
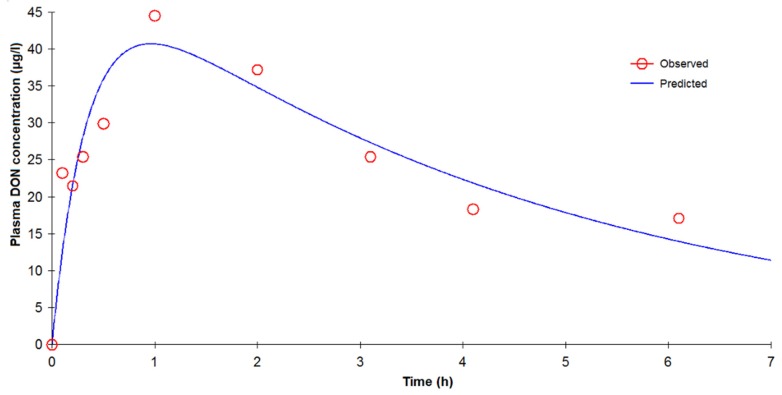
One-compartment model fitted to DON concentrations (µg/L) *vs.* time (h) in plasma in a representative pig without t lag after oral administration of 100 µg/kg of DON.

After oral administration of DON, the plasma concentration *vs.* time curves in pigs were best described by a one-compartment model with first order absorption and elimination without a lag time (Figure 3) based on the following equation:

(2) $$C\left(t\right)=\frac{FDK_{01}}{V\left(K_{01}-K_{10}\right)}[e^{-K_{10}\left(t\right)}-e^{-K_{01}\left(t\right)}$$

where *F* is the bioavailability, *D* the dose; *K*_01_ the rate of absorption, *V* the apparent volume of distribution and *K*_10_ the rate of elimination. Table 3 presents the kinetic parameters obtained from Equation (5).

**Table 3:** Individual and mean toxicokinetic parameters of DON estimated from a one-compartment model in the plasma of seven pigs following oral administration of a single dose of 100 µg/kg.

Parameters (Units)	1	2	3	4	5	6	7	Mean	SD
***V*/*F* (L/kg)**	1.63	2.66	1.51	1.04	2.58	1.85	2.77	2.01	0.67
***K*_01_ (1/h)**	2.86	5.22	13.99	0.43	0.85	0.53	2.20	3.72	4.83
***K*_10_ (1/h)**	0.22	0.10	0.38	0.43	0.13	0.50	0.30	0.29	0.15
**AUC_INF_ (h·µg/L)**	225.12	319.67	198.20	197.36	256.97	127.21	110.66	205.03	72.24
***t*_1/2_*K*_01_ (h)**	0.24	0.13	0.052	1.63	0.82	1.30	0.32	0.19 ^a^	0.38
***t*_1/2_*K*_10_ (h)**	3.09	6.62	2.46	1.62	5.28	1.39	2.34	2.47 ^a^	1.32
***CL*/*F* L/h/kg)**	0.37	0.28	0.45	0.44	0.34	0.92	0.82	0.52	0.25
***t*_max_ (h)**	0.97	0.76	0.30	2.34	2.60	1.94	1.05	1.42	0.87
***C*max (µg/L)**	40.67	30.91	51.31	31.02	23.97	24.09	23.98	32.28	10.34

*V*/*F*: volume of distribution divided by bioavailability (F); *K*_01_: absorption rate constant; *K*_10_: elimination rate constant; AUC: area under the curve; *t*_1/2_: biological half-life of *K_01_* (absorption) or *K*_10_ (elimination); *t*_max_: time of maximum plasma DON concentration; *C*_max_: maximum plasma DON level. ^a^: harmonic mean.

The mean half-life of the elimination phase was established at 2.47 ± 1.32 h. No statistical difference was found between the elimination half-life obtained by IV or oral routes. The peak concentration (*C*_max_) of 23.97–51.31 µg/L plasma was reached (*t*_max_) between 0.30 and 2.60 h (Table 3). The mean half-life of absorption (0.19 ± 0.38 h) and the *t*_max_ value show rapid absorption. Table 2 shows that the MRT was 5.59 ± 4.01 h.

Table 4 shows a general overview of mean bioavailability estimated by compartmental, non-compartmental, and deconvolution approaches. For pigs, bioavailability was estimated at 84.44% ± 33.98% on the basis of parameters obtained after modeling the concentration profile. NCA analysis estimated bioavailability at 83.99% ± 48.59%. For gavage, the AUC extrapolated between the last point and infinity to estimate total AUC (AUC_INF_) contributed over 20% (Table 2) [22]. Consequently, we also calculated AUC from 0 to the last point (AUC_last_). With this parameter, bioavailability was estimated at 58.3% ± 25.6%. Deconvolution analysis estimated absolute bioavailability at 70.5% ± 25.6%. No statistical difference was observed for bioavailability obtained by modeling, NCA or deconvolution analysis.

**Table 4:** Comparison of mean bioavailability of DON in rats (*n* = 3 by sampling time) and pigs (*n* = 7) after modeling, NCA, and deconvolution.

Animal model	Bioavailability (%)			
	Modeling	NCA		Deconvolution
		INF ^a^	Last ^b^	
**Pigs**	84.4 ± 34.0	84.0 ± 48.6	58.3 ± 25.6	70.5 ± 25.6
**Rats**	-	126.3	47.3	-

^a^ bioavailability calculated with AUC_INF_; ^b^ bioavailability calculated with AUC_last_.

**Figure 4:**
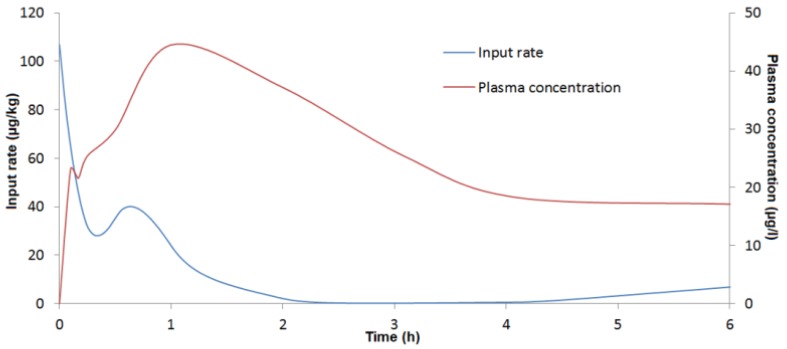
Time course evolution of DON concentrations in plasma and input rate estimated (µg/kg) from deconvolution analysis in a representative pig after oral administration of 100 µg/kg of DON.

The presence of double peaks on all DON concentration curves for pig plasma after oral administration and at the beginning of kinetic analysis suggested non-continuous absorption phases in all pigs. Deconvolution analysis confirmed the presence of double absorption as presented in Figure 4 and reported in Table 5. The first absorption phase lasted 0.32 ± 0.12 h and represented close to 25% of global absorption (71%). The second absorption phase was bigger (46%) and 10 times longer (4.61 ± 1.56 h). 

**Table 5:** Determination of the duration and percentage of absorption after deconvolution analysis of concentration profiles following oral administration of 100 µg/kg of DON in pigs.

Deconvolution analysis	Animal Number							Mean	SD
	1	2	3	4	5	6	7		
**Duration 1st phase (h)**	0.37	0.24	0.32	0.18	0.55	0.3	0.29	0.32	0.117
**Absorption 1st phase (%)**	22.6	21.9	54.0	8.8	43.0	12.8	9.9	24.7	17.4
**Duration 2nd phase (h)**	5.73	3.76	1.76	5.82	5.67	5.7	3.81	4.61	1.555
**Absorption 2nd phase (%)**	47.6	33.4	23.0	104.4	37.7	55.2	19.2	45.8	28.8

In order to evaluate the accumulation of DON, subchronic DON exposure (100 µg/kg·bw) was performed with DON-contaminated diets for three days. Pig plasma concentrations of DON obtained from subchronic DON exposure with contaminated diets lay below the method’s LOQ. Furthermore, DON was not detected in the fecal samples analyzed.

For gavage of rats, 70% of AUC_INF_ was extrapolated for gavage, so the value of bioavailability to retain is the value estimated from AUC_last_. The AUC_last_ value was 14.63 ± 4.45 h·µg/L and the corresponding bioavailability was estimated at 47.3%, ranging from 45.1% to 49.2%. Peak concentrations (*C*_max_) of 10.44 ± 3.39 µg/L of DON were reached (*t*_max_) at 0.17 h, indicating rapid absorption. MRT_last_ was established at 0.95 h.

## 3. Discussion

This study assessed DON toxicokinetics in two animal models after intravenous (IV) administration and oral gavage at the NOAEL.

Our clinical observations were in accordance with Pestka *et al.* (1987), who reported that 50 µg/kg live weight of DON was the minimum effective dose that provoked emesis in pigs [23]. Only IV administration provokes emesis due to the rapid distribution of DON and a high concentration in the brain, leading to a central action by major neurotransmitters such as noradrenaline, dopamine, or serotonin [14,24].

For the toxicokinetic study, we only focused on plasma concentrations of DON because not as many metabolites of DON are present in pigs as the parent molecule [25], although glucuronide should not be neglected [26]. However, DON toxicity is mainly due to the parent molecule. Indeed, in most cases, metabolites are a detoxification product of DON and appear less harmful [27,28,29]. They are unable to give DON back into the organism, unlike DON-3-Glucoside [30]. After IV administration of DON in pigs, Prelusky *et al.* (1988) adapted a two- and three-compartment model, whereas Goyarts and Danicke (2006) described a two-compartment model as shown in the current study [17,25]. From our results and these observations, it could be suggested that the model’s fit depends on the frequency of blood sampling during the initial minutes after dosing as well as the performance of the analytical methods used, and in particular the limit of quantification. Prelusky *et al.* (1988) analyzed the disappearance of radioactivity in plasma after application of ^14^C-labelled DON, but revised this method because of inaccuracy at low doses [16,17]. The toxicokinetic parameters determined in our study indicate that DON was not widely distributed and was rapidly eliminated from plasma. These observations suggest that there is no accumulation in tissues as proposed by Prelusky *et al.* (1988) [17]. However, a comparison with the results from previous studies is not possible because not all sample results had levels above the LOQ of the method used. 

In contrast to pigs, information on the IV toxicokinetic parameters of DON in rats is scarce. Following the IV administration to rats, we observed through a sparse sampling procedure that extrapolated data made a large contribution to the estimation of AUC_INF_. Consequently, only the parameters estimated between 0 and the last point above the LOQ were considered. Taking this point into account, the two animal species showed major differences, as AUC_last_ was seven-fold higher in pigs than in rats. After the administration of 100 µg/kg·bw of DON, the plasma concentrations were much higher in pigs than in rats. This observation may be explained by different metabolization velocities that have been described earlier. Maul *et al.* (2015) investigated DON metabolism using liver microsomes from various animal species [31]. They highlighted that liver microsomes from rats led to relatively rapid apparent intrinsic clearance of DON by glucuronidation, while liver microsomes from pigs showed more moderate activity.

After oral administration in pigs, the elimination rate (2.47 h) is fairly similar to the elimination rate following IV administration (1.53 h). This result is not in accordance with Goyarts and Danicke (2006) [14], but could be explained by the difference in DON administration route (diet *vs.* oral gavage). Furthermore, mean half-life should be calculated using a harmonic mean as recommended by Lam *et al.* (1985) and not the classic arithmetic mean used by Goyarts and Danicke (2006) [14,32]. By using a harmonic mean on Goyarts’ data, no difference was observed. Absorption is very rapid (half-life close to 15 min) and in accordance with the rapid appearance (*t*_max_ 0.3 to 2.6 h) of the plasma’s peak DON concentration (*C*_max_). These results lie within the same range reported in the literature. Following an intragastric application of 600 µg/kg of DON, Prelusky *et al.* reported (1988) a peak concentration within 15–30 min with a maximal plasma concentration range for DON of 63–325 ng/mL) [17]. In another toxicokinetic study in pigs, Prelusky *et al.* (1990) measured maximal DON concentrations (*C*_max_) of 367 ± 37 µg/L after 3.75 ± 1.26 h (*t*_max_) following intragastric administration of 1000 µg/kg of DON [16].

DON was also rapidly absorbed by rats, with maximal plasma concentration 0.17 h after oral administration. In mice, Pestka *et al.* (2008) reported two-compartment toxicokinetics with a *C*_max_ of 12 µg/mL within 5–15 min following oral administration of 25 mg/kg·bw of DON [19]. These results are in agreement with our toxicokinetic study of DON in rats. The latter exhibited a lower *C*_max_ than pigs (1:4) while the AUC_last_ was eight times lower.

To our knowledge, this is the first study comparing oral bioavailability of DON in two different animal models at the same dose. The bioavailability of DON appeared higher in pigs than in rats. However, in rats, the absolute bioavailability value could not be taken into consideration because of the major contribution of extrapolation in AUC_INF_ calculation. The low bioavailability of rats was estimated at 47%, suggesting that more than half the DON dose remained in the rats’ intestines. When compared to the bioavailability of DON in pigs calculated with AUC_last_ (58%), this difference could indicate that DON has a larger impact on the intestinal microbiota of rats than pigs, as already reported in the literature [33,34]. The data of the present study may also be interpreted as it being the fast metabolism which may cause low bioavailability of free DON. For example, Schwartz-Zimmermann *et al.* (2014) have recently highlighted the whole spectrum of DON metabolites in rats in a feeding trial. The quantitation of DON and its sulfonates in rat feces revealed that DON sulfonates accounted for approximately 50% of the total amount of DON administered [35]. These rat trial results notably revealed the near exclusive occurrence of DON sulfonates in feces and indicated formation of sulfonates in the gut intestinal tract. DON sulfonate formation could lower the absorption of DON and explain the difference in bioavailability between rats and pigs.

The absolute bioavailability of DON in pigs (70%) estimated in this study was in accordance with the results of Prelusky *et al.* (1988) and Goyarts and Danicke (2006), who found a bioavailability value of 54.8% ± 8.6% and 54.1% ± 17.5%, respectively [14,17]. As absolute bioavailability was determined after data modelling, values may be incorrectly estimated because the model did not fit the data well. Our study, for instance, did not take into account the presence of the double peak. This hypothesis is supported by the fact that the bioavailability reported by the abovementioned authors is closer to the value we obtained from AUC_last_ calculation than from our deconvolution analysis. In contrast, no double peak was observed during the absorption phase in rats, which could be due to the frequency of blood sampling during the initial minutes after dosing. This double peak, systematically observed in all pigs during the absorption phase, indicates that absorption could start in the stomach or upper part of the duodenum (first peak) as suggested by Goyarts *et al.* (2006) and assumed by the findings of Eriksen *et al.* (2003) and Danicke *et al.* (2004) [14,25,27]. A second, larger portion of the dose passes into the blood (second peak) from the intestine. The presence of these discontinuous biphasic absorption patterns could not be related to a food effect because the pigs were fed at least 4 h after DON administration. Further studies are needed to assess the clinical significance of these findings. In addition, the double absorption peak observed could explain the capacity of DON to induce emesis after oral exposure. The minimum emetic plasma concentration of DON can be reached with a rapid, high initial absorption peak.

From a biological point of view, the absence of DON in pig feces could be explained by its high absorption in the small intestine and the greater excretion of free DON and its derivatives in urine (68%) than in feces (20%) [14,25,36]. Moreover, some bacteria belonging to the gut microbiota are also known to play a role in the detoxification of native DON in the colon, inducing a lower amount of DON in fecal samples [37]. From an analytical point of view, the level of DON in feces could be undetectable by the method used.

It would have been advantageous to obtain a full excretion profile of DON and its metabolites (DOM-1, DON-3/15-Glucuronide and DON sulfonates) to evaluate the behavior of DON in both animal species and compare it in humans. This is especially important as systemic exposure does not only relate to the absorption phase: DON metabolism could be equally important, and should thus be taken into consideration in further experiments. A good animal model should resemble humans in terms of both bioavailability and metabolic profile. From our findings, the two animal models could be useful in two different ways in the framework of DON risk assessment with a high-exposure scenario. On the one hand, the high bioavailability of pigs could make them a better experimental model for toxicological studies than rats. On the other, the low bioavailability of rats makes them a better model for studying the impact of DON on intestinal microbiota considering that a higher part of the dose ingested may remain in the intestine, as observed in rats and in human microbiota-associated rats [33,38].

## 4. Experimental Section

### 4.1. Animals

All animal procedures were carried out in strict accordance with the recommendations of the French Ministry of Agriculture. The protocol was approved by ANSES’s Committee for Ethical Standards and performed in our approved animal breeding facility (Permit Number: D35-137-26). 

Female Sprague-Dawley rats catheterized in the jugular vein were obtained from the breeding facility of Janvier Labs (Saint Berthevin, France). Pelleted feed free of mycotoxin contamination (SAFE, Scientific Animal Food and Engineering, Augy, France) and water were provided *ad libitum*. Twenty-one catheterized rats (eight weeks old, 120–150 g·bw) were housed by three in a polycarbonate cage. Large White Landrace Pietrain sows were obtained from the breeding facility of INRA, France’s national institute for agricultural research (INRA, Saint-Gilles, France). Pelleted feed free of mycotoxin contamination (Cooperl Arc Atlantique, Vitré, France) was distributed twice daily in two equal meals (600 g), while water was provided *ad libitum*. Seven pigs (26–28 kg·bw) were housed individually. Animals were acclimatized for one week.

### 4.2. Surgery

To facilitate blood collection, the pigs were cannulated at the jugular vein. This surgery was performed under sterile conditions. The pigs were pre-anesthetized by an intramuscular application of a mixture of xylazin (0.1 mL/kg·bw) (Rompun^®^ 2%, 20 mg/mL, Bayer HealthCare, Monheim, Germany) and ketamin (0.1 mL/kg bw) (Imalgène^®^ 1000, 50 mg/mL, Merial, Lyon, France). Pre-anesthetized pigs received 0.04 mL/kg·bw of atropine (atropine sulfate aguettant^®^, 1 mg/mL, Aguettant, Lyon, France) subcutaneously. Endotracheal intubation was performed and the tube (2 mm diameter) was then connected to a large animal anesthetic circle system equipped with a mechanical ventilator (Parker Hannifin, Contamine-sur-Arve, France). The anesthesia was maintained by administration of isofluran (Aerrane^®^, Baxter S.A., Maurepas, France) in pure oxygen (2 L/min) during surgery. Two sterile catheters purchased from VWR (Strasbourg, France) were used for cannulation of the jugular vein. One was for collecting blood samples (1.02 mm × 2.16 mm, 80 cm, No. 28-0148) and the other for administering DON (0.76 mm × 1.65 mm, 80 cm, No. 28-0147). The catheters were fixed with ligatures in the jugular vein and tunneled subcutaneously, exteriorized dorsally in the neck and fixed at the skin. A heparinized physiological saline solution (B. Braun Avitum^®^, Gradignan, France) was used to maintain catheter patency. Animals were allowed to recover for at least three days before the kinetic study was initiated.

### 4.3. Chemicals, Products, and Reagents

Deoxynivalenol was purchased from Sigma-Aldrich (Saint-Quentin Fallavier, France) and dissolved in acetonitrile HPLC grade (Sigma-Aldrich) at 1 mg/mL. This solution was stored for a maximum of one year at −18 °C according to the manufacturer’s instructions. The administration solutions used in the kinetic studies were diluted in physiological saline solution (B. Braun Avitum) on the day of the experiment. Methanol (analytical reagent grade) was purchased from Fisher Scientific (Illkirch, France). Oasis^®^ HLB 30 mg and Sep-Pak^®^ C18 100 mg cartridges were obtained from Waters (St Quentin-en-Yvelines, France). Mycosep 227 Trich + columns were purchased from Romer Labs^®^ Diagnostic (Tulln, Austria). The Millex-HV syringe filter, PVDF, 0.45 µm came from Millipore (Molsheim, France).

### 4.4. Study Design

#### 4.4.1. Pigs

The animal experiments were performed as per a two-way cross-over design. DON solutions were administered at 100 µg/kg·bw intravenously (IV) or orally (PO). Four animals were given an oral bolus and three received the mycotoxin intravenously. After a wash-out period of one week, animals that had previously been given an oral DON bolus received an intravenous bolus and *vice versa*. Blood samples were collected from each pig before then at 1 min, 2 min, 4 min, 8 min, 15 min, 30 min, 1 h, 2 h, 3 h, 4 h, 6 h, 8 h, 10 h, and 24 h after IV administration; and before then at 5 min, 10 min, 15 min, 30 min, 1 h, 2 h, 3 h, 4 h, 6 h, 10 h, and 24 h after oral administration in heparinized tubes (10 UI of heparin per mL). In order to evaluate the accumulation of DON, subchronic DON exposure (100 µg/kg·bw) was performed with DON-contaminated diets for three days. Blood samples were collected from each pig on day 1 before and at 5 min, 15 min, 30 min, 1 h, 2 h, 3 h, 4 h, 6 h, 10 h, 24 h; on day 2 at 15 min, 1 h, 2 h, 4 h, 6 h, 24 h; and on day 3 at 15 min, 1 h, 2 h, 3 h, 4 h, 6 h, 10 h, 24 h, and 48 h after administration. Individual fecal samples were collected 6 h after DON administration every day for three days and stored at −20 °C until analysis.

The blood samples (approximately 5 mL) collected were centrifuged (3000× g for 10 min) and the plasma stored at −20 °C until analysis.

#### 4.4.2. Rats

As a smaller amount of blood can be collected from rodents than pigs, the kinetic study was carried out according to a sparse sampling protocol, in which each rat was sampled only once per route of administration. Three animals were sampled per time point. A solution of DON was administered at the dose of 100 µg/kg bw intravenously (IV) and orally (PO) after a wash-out period of one week. Blood samples were collected at 0 min, 1 min, 4 min, 8 min, 30 min, 1 h, 3 h, 6 h after IV administration, and at 0 min, 10 min, 30 min, 1 h, 2 h, 3 h, 6 h after oral administration in heparinized tubes (10 UI of heparin per mL). The blood samples (approximately 1 mL) collected were centrifuged (3000× *g* for 10 min) and the plasma stored at −20 °C until analysis. Individual rectal fecal samples were collected at 6 h and 24 h after DON administration. Samples were stored at −20 °C until analysis.

### 4.5. Determination of DON Concentration

#### 4.5.1. Plasma Analysis

Plasma samples were analyzed for DON by an in-house high-performance liquid chromatography (HPLC) method with ultraviolet detection (UV).

##### Sample Preparation

For pig plasma, 0.5 mL of sample was directly applied on an SPE Oasis^®^ HLB cartridge. The column was washed once with 1 mL of water, then once with 0.5 mL of water/methanol (80/20, *v*/*v*). Once the cartridge was dried, DON was eluted using 0.5 mL of acetonitrile. The eluate was evaporated using a gentle nitrogen (N2) stream (~45 °C). The dry residue was reconstituted in 0.25 mL of ultra-pure water (Milli-Q system, Millipore, Molsheim, France).

For rat plasma, proteins were first precipitated by adding two volumes of a water/methanol mixture (80/20, *v*/*v*) to one volume of plasma. The samples were stored for 15 min at +4 °C, followed by a centrifugation step (3000× *g* for 10 min). The supernatant was then applied to an SPE cartridge and prepared as described previously for pig plasma. 

After vortex mixing, samples were transferred to autosampler vials and injected into the HPLC instrument.

##### Chromatography Conditions

The HPLC system consisted of an Agilent Technologies series 1100 (Les Ulis, France), equipped with a diode array detector. Chromatographic separation was performed on a Lichrospher^®^ 100 RP-18 endcapped (5 µm) column (125× 4 mm) (Merck Millipore, Molsheim, France) with detection set at 219 nm. The mobile phase consisted of a linear gradient of water (A) and acetonitrile (B). The proportion of B was increased from 2% (0 min) to 15% (3 min), kept steady for 7.5 min, then the column was re-equilibrated for 5 min under initial conditions. All separations were carried out at 25 °C with a flow rate of 0.8 mL/min. 

#### 4.5.2. Fecal Analysis

HPLC-UV was insufficient to detect and quantify DON in feces, so fecal samples were further analyzed by a liquid chromatography-tandem mass spectrometry (LC-MS/MS) method adapted from laboratory LDA 22 (Ploufragan, France).

##### Sample Preparation

For pig feces, DON was extracted by adding 1 mL of water and 8.4 mL of acetonitrile to 1 g of sample. After stirring (15 min) and centrifugation (5000× *g* for 10 min), the supernatant was purified with a Mycosep column according to the manufacturer’s instructions, then 2 mL was evaporated under a nitrogen stream (~45 °C). Dry residue was reconstituted in 0.2 mL of ultra-pure water and filtered with a Millex^®^ unit (Merck Millipore) before being injected into the LC instrument.

For rat feces, 5 mL of water was added to 0.5 g of sample. After stirring (15 min) and centrifugation (20,000× *g* for 10 min), the supernatant was purified using a C18 Sep-Pack cartridge. After washing with 2 mL of water, DON was eluted with 1 mL of acetonitrile. The residue was evaporated under a nitrogen stream, then dissolved in 0.2 mL of water and filtered with a Millex^®^ unit before being injected into the LC instrument. A volume of 100 µL of sample was injected into the chromatography system.

##### Chromatography Conditions

Liquid chromatography was performed using an Ultimate 3000 LC (Thermo Fisher Scientific, Villebon-sur-Yvette, France). DON was separated using a Zorbax Eclipse XDB C8 column (150× 2 mm, 5 µm particle size) at a flow rate of 0.2 mL/min. A volume of 10 µL was injected. The mobile phase consisted of a linear gradient of acetic acid 0.1% and acetonitrile. The proportion of acetonitrile increased from 2% (0 min) to 50% (5 min), and then the column was re-equilibrated for 5 min under initial conditions. The retention time of the analyte was 4.9 min.

The HPLC system was coupled to a Triple Stage Quadrupole (TSQ) Vantage mass spectrometer (Thermo Scientific) with the electrospray ionization (ESI) source set to negative mode. Two SRM transitions (*m*/*z*) were monitored for DON *i.e.*, *m*/*z* [M + CH3COO]-: 355.0 > 295.1 and 355.0 > 265.1. The following settings were used: ion spray voltage 3500 V, collision energy 10 eV, vaporizer temperature 300 °C, tube lens voltage at 60 V, sheath and auxiliary gas pressure 40 and 35 psi, respectively. The XCalibur v2.1 software (Thermo Scientific) was used for system control and data processing.

#### 4.5.3. In-House Method Validation

Validation studies were performed using calibration curves and matrix-matched validation samples. For pigs, five levels of concentration were used, repeated three times and for three runs *i.e.*, a total number of 45 spiked samples. For rats, four levels of concentration were used, repeated three times and for two runs *i.e.*, a total number of 24 spiked samples. Methods were validated using an approach based on accuracy profiles, composed of trueness and precision. The validation data were processed by e-noval v3 (Arlenda, Liege, Belgium).

For plasma, trueness was expressed in terms of recovery and the method’s mean recovery was 90.5% in pigs and 97.9% in rats. Precision was evaluated by repeatability and intermediate precision at each concentration level. Repeatability and intermediate precision values were acceptable for toxicokinetic studies since they lay between 3.3% and 7.6% for pig plasma and between 1.8% and 11.1% for rat plasma. 

The dosage range to determine the upper and lower limits of quantification went from 5 to 250 ng/mL for pig plasma and from 5 to 100 ng/mL for rat plasma. DON limits of quantification (LOQs) were established at 6.7–247.5 ng/mL and 5.2–99 ng/mL in pig and rat plasma, respectively.

For pig feces, the dosage range used went from 10 to 500 ng/g and defined the lowest and highest LOQs. The method’s mean recovery was 97.92% and 88.75% for mass transition 1 (295) and 2 (265), respectively. Repeatability and intermediate precision values lay between 2.0% and 5.4% for transition 1% and 0.7% and 3.8% for transition 2. 

For rat feces, it was not possible to validate the method because precision was unacceptable.

### 4.6. Kinetic Analysis

For all the studies involved, concentrations under the LOQ were not retained for calculations. Both compartmental and non-compartmental approaches were used to determine the kinetic parameters. All plasma concentration *vs.* time curve analyses were performed with Phoenix WinNonlin 6.3 software (Certara, Saint Louis, MO, USA).

#### 4.6.1. Compartmental Analysis

For pigs, two- and three-compartment models were tested for the intravenous route, whereas one- and two-compartment models (with and without a lag time) were compared for oral administration. Models were compared and evaluated by application of Akaike’s Information Criterion [20].

#### 4.6.2. Non-Compartmental Analysis (NCA)

The total area under the plasma *vs.* time curve (AUC) for DON was determined using the linear trapezoidal rule with extrapolation to infinity. Extrapolation AUC_(last-INF)_ was based on the following equation:

(3) $$AUC_{\left(last-INF\right)}=\frac{C_{last}}{\lambda_{\text{z}}}$$

where *C*_last_ is the last quantifiable plasma concentration and λ_z_ the slope of the terminal phase. The terminal slope was estimated from the linear part of the terminal phase by at least three points and was accepted with a coefficient of determination (*r*^2^ > 0.95). The Mean Residence Time (MRT) was calculated using the linear trapezoidal rule between 0 and *C*_last_ or with extrapolation to infinity. Clearance and Vss *i.e.*, the volume of distribution at steady state, were also estimated.

For oral administration (po), the observed maximum concentration (*C*_max_) and the corresponding time (*t*_max_) were obtained from the concentration *vs.* time profile and compared to those obtained from compartmental analysis. The bioavailability factor was defined according to the formula below:

(4) $$F=\frac{{\left(AUC\right)}_{p.o.}\times {\left(Dose\right)}_{i.v.}}{{\left(AUC\right)}_{i.v.}\times {\left(Dose\right)}_{p.o.}}\times 100$$

where AUC*_p.o._* or *_i.v._* represents the area under concentration *vs.* time curve 0 to the last quantifiable concentration (AUC_last_) or 0 to infinity (AUC_INF_) after oral or intravenous administration of DON. Dose*_p.o._* or *_i.v._* represents the actual dose administered by oral or intravenous routes.

For rats, sparse data analysis was chosen because we only had one point per animal and three animals per time point. In this kind of study, it was not possible to distinguish inter-individual from intra-individual variability, and consequently the present analysis focused on mean parameters and not on inter-individual variability.

#### 4.6.3. Deconvolution Analysis

To assess absolute bioavailability and the *in vivo* input rate of DON, we undertook deconvolution analysis. This tool has been widely used to assess the gastrointestinal absorption of prodrugs in pigs [39] or the secretory profile of hormones [40]. Briefly, deconvolution gives an estimation of the drug input rate over time using the unit impulse response function (UIR) by a convolution equation:

(5) $$c\left(t\right)=\int_{0}^{t}f\left(t-u\right)c_{\partial }\left(u\right)du\equiv f\left(t\right)*c_{\partial }\left(t\right)$$

where * denotes the convolution operator, *f*(*t*) is the UIR *i.e.*, the disposition function and *c*_δ_(*t*) the drug input rate.

By deconvolving *c*(*t*) with *f*(*t*), an *in vivo* DON input rate can be determined. The disposition function was obtained from the toxicokinetic parameters of DON after IV administration.

### 4.7. Statistical Analysis

Toxicokinetic parameters are expressed as an arithmetic mean and its standard deviation, except for half-lives where harmonic mean and its standard deviation were calculated [32]. The toxicokinetic parameters of rats and pigs (AUC, *C*_max_) were compared after IV and oral administration by a *t* test or a non-parametric test (Mann-Whitney test) when the variance was not homogeneous. For pigs, the elimination half-life obtained after IV and oral administration was also compared by a *t* test. For bioavailability obtained from compartmental, non-compartmental, and deconvolution approaches, a Levene’s test was used to verify the homogeneity of variance followed by a one-way ANOVA. A level of significance of 0.05 was retained. All statistical analyses were carried out using SYSTAT v13 software (Systat Software, Chicago, IL, USA).

## 5. Conclusions

In this work, by using deconvolution analysis to assess the absolute bioavailability of DON, we were able to provide more reliable and recent data on this important kinetic parameter. We also showed important differences between pigs and rats that should be taken into account for risk assessment.
